# Optimizing Healthcare Through Digital Health and Wellness Solutions to Meet the Needs of Patients With Chronic Disease During the COVID-19 Era

**DOI:** 10.3389/fpubh.2021.667654

**Published:** 2021-07-12

**Authors:** Azizi A. Seixas, Iredia M. Olaye, Stephen P. Wall, Pat Dunn

**Affiliations:** ^1^Department of Population Health, Department of Psychiatry, New York University (NYU) Grossman School of Medicine, New York, NY, United States; ^2^Department of Medicine Division of Clinical Epidemiology and Evaluative Sciences Research, Weill Cornell Medical College, New York, NY, United States; ^3^Department of Emergency Medicine, Department of Population Health, NYU Grossman School of Medicine, New York, NY, United States; ^4^American Heart Association, Center for Health Technology and Innovation, New York, NY, United States

**Keywords:** COVID-19, gamification, digital health, chronic condition, digital health (eHealth)

## Abstract

The COVID-19 pandemic exposed and exacerbated longstanding inefficiencies and deficiencies in chronic disease management and treatment in the United States, such as a fragmented healthcare experience and system, narrowly focused services, limited resources beyond office visits, expensive yet low quality care, and poor access to comprehensive prevention and non-pharmacological resources. It is feared that the addition of COVID-19 survivors to the pool of chronic disease patients will burden an already precarious healthcare system struggling to meet the needs of chronic disease patients. Digital health and telemedicine solutions, which exploded during the pandemic, may address many inefficiencies and deficiencies in chronic disease management, such as increasing access to care. However, these solutions are not panaceas as they are replete with several limitations, such as low uptake, poor engagement, and low long-term use. To fully optimize digital health and telemedicine solutions, we argue for the gamification of digital health and telemedicine solutions through a pantheoretical framework—one that uses personalized, contextualized, and behavioral science algorithms, data, evidence, and theories to ground treatments.

## Chronic Disease Care Management in the Pre-COVID Era

The novel coronavirus disease (COVID-19) pandemic has exposed longstanding and foundational fractures, inefficiencies, deficiencies, and inequities in the American healthcare system, most notably how we deliver healthcare and manage chronic diseases. COVID-19 exposed known truths about the U.S. healthcare delivery and management systems as being rigid, intermittent, fragmented, inaccessible, antiquated in parts, impersonal, and unable to accommodate complexity and high volume without compromising the quality of care. Although the management of COVID-19 was initially poor, the management of chronic diseases during the same period was worse, with 41% of US adults avoiding some form of medical care out of fear of contracting COVID-19 (1). Initial autopsy of why the chronic disease management infrastructure crumbled during the COVID-19 pandemic revealed that the surge of COVID patients consumed all the resources of health systems leaving insufficient medical resources for chronic disease patients. Most health systems were unable to meet the basic needs of chronic disease patients, many of whom were not even receiving sufficient care and support from the health system, pre-COVID. Chronic disease management became even more dire in order to meet the high burden of: 1. COVID-19 infections and deaths, 2. COVID-19 survivors who are at greater risk for a chronic disease, and 3. individuals with chronic disease, pre-COVID. The medical needs of these three groups of patients are so great that there is fear they may break healthcare's levee systems, a brick-and-mortar system that provides fragmented, infrequent, and incremental visit-to-visit care. These fears are substantiated by the high prevalence (six out of 10) of Americans with a chronic disease and it is estimated that COVID-19 survivors who are at increased risk of developing chronic health conditions and diseases will increase the prevalence of chronic disease (2–4).

The high prevalence of non-COVID and COVID-related chronic diseases will place even greater burden on a precarious chronic disease management healthcare infrastructure (5). Low income and underserved patients are most vulnerable to the inadequacies of the current chronic disease management infrastructure, as they are less likely receive basic care and management (6). It is therefore an imperative to urgently develop and implement innovative and personalized strategies to optimize chronic disease care for all, especially for disparity communities (7–10).

## Advantages and Disadvantages of Telemedicine in Chronic Disease Management

The increased demand for remote health services due to COVID-19 spurred the largest and fastest implementation and utilization of telemedicine and digital health solutions in the US healthcare system. For example, in fields like hepatology and gastroenterology, telemedicine usage increased by 4,000% (11). Although telemedicine and digital solutions cannot completely replace all components of the healthcare experience, there are some unique advantages, such as convenience of care, face-to-face interaction, and increased access to care, which was crucial for some medical fields like mental health services (12, 13). Telemedicine and digital health solutions also increased access to healthcare among demographic groups that have historically low utilization of healthcare services. Black patients were more likely to receive diagnosis for a chronic condition *via* a telemedicine visit than in-person. Additionally evidence highlight that telemedicine and digital health solutions have no compromised quality of care. In one study, telemedicine and in-person appointments had the same patient satisfaction (ranging from 94 to 99% satisfaction), with telemedicine rated as significantly more convenient (14). Overall, telemedicine has allowed providers to virtually deliver high-quality healthcare at a faster rate and provide more convenient wellness visits for patients.

Although, the burgeoning of telemedicine is widely seen as a positive step to improve access, delivery of healthcare, and management of disease, it does not solve some of the fundamental flaws of the delivery and management system, such as the lack of seamless patient experience and wraparound and continuous support to patients to better self-manage chronic disease. Despite the promise of telemedicine, early evidence from March 2020-May 2020 suggest that it is not a panacea that cures all the ills of a broken healthcare system, as almost 50% of US adults avoided medical care during the pandemic (1). Of the missed medical care appointments, 31.5% were routine and 12% were urgent/emergency. Individuals who missed medical care primarily: (1) had two or more underlying chronic health conditions (55%), (2) had no medical coverage (42%), (3) identified as Hispanic/Latinx (55%) or Black (48%), and (4) considered high risk (49%) (1). These evidence suggest that the delivery of telemedicine, as currently constructed, is not suited for individuals with a chronic disease, and as such the delivery of telemedicine and digital health must be refined and customized to serve these individuals.

## The Need for Digital Health and Wellness Applications in Telemedicine to Support Chronic Disease Care Management

Patients with chronic disease need more than discrete and infrequent health checkups through telemedicine and instead need consistent engagement as well as wrap-around and continuous support (15). While the rapid pivot to telemedicine and digital tools was an adjustment to patients and clinicians alike, it has also served as a lifeline to patients with chronic conditions. For example, many cardiac rehabilitation programs had to shut down due to social distancing practices. Providing such resources, services and infrastructure has to be remote, seamless, frictionless, and well-connected. If healthcare does not transform quickly to meet the needs of individuals with chronic diseases during this COVID era, more people will die at accelerated rates. According to the CDC and USAHearts.org 600,000 individuals died in the United States from heart disease or cancer during the period February 1 to June 27th, 2020. Based on these facts, “in-game” halftime adjustments need to be made to stem the tide of excess mortality due to poor and limited management of chronic disease patients.

In order to improve healthcare delivery through innovation, health systems must: (1) ensure and optimize patient use, engagement, attention, and motivation to adhere to treatment regimens, and (2) address provider and system-level barriers to care and mitigate effects of social determinants of health (16). The ubiquitous and proliferative use of digital health, patient-generated health data devices, and telemedicine provides the ideal suite of tools and terroir to revolutionize how healthcare evaluates, diagnoses, treats and manages patients in the wake of the COVID-19 pandemic. Telemedicine and digital health can complement the contemporaneous modification of our healthcare system which has been transformed from an in-person brick-and-mortar management system into an asynchronous and synchronous virtual care system through the burgeoning of telehealth. Although telemedicine and digital health have limited the amount of in-person ambulatory care, there is still a need to provide (1) real-time and convenient tracking of health parameters and behaviors; (2) flexible individualized and adaptive treatment and management programs, and (3) continuous support to manage health and wellness. Despite the promise and perils of telemedicine and digital health solutions, early evidence suggest that they are underutilized due to poor engagement, uptake, and long-term use and adherence (17).

## Improving Engagement in and Adherence to Digital Health Solutions

We argue that gamifying, contextualizing, and personalizing the telemedicine and digital health experience of users can improve engagement, uptake, long-term use, and adherence to digital health solutions. Specifically, we argue that the use of a pantheoretical framework can optimize engagement and adherence to digital health solutions. A pantheoretical approach uses: (1) top-down (the use of theory to deduce and design a health and wellness intervention) and (2) bottom-up (the use of data to determine general pattern recognitions to create, modify, and inform components of digital health interventions) evidence and approaches to personalize health and wellness interventions and treatments based on provider, psychosocial, system, behavioral, biological, clinical, and environmental data. Pantheoretical approaches, which can be categorized as part of a suite of innovative behavioral approaches such as just-in-time adaptive interventions (18–20), adds a unique workflow and evidence for achieving more ecologically valid approaches to overcome barriers and accentuate facilitators of engagement and adherence to telemedicine and digital solutions. The pantheoretical workflow includes four steps: tailoring, clustering/profiling, personalizing and optimizing interventions/strategies, which uses modifiable and non-modifiable factors as well as nomothetic (general) and idiographic (personalized) approaches, to personalize behavioral and lifestyle modification strategies and treatment adherence (21). Greater detail of the pantheoretical approach is described in our seminal paper published in 2020 (21).

### Gamification

Gamification, which is just one strategy of many to improve adherence and engagement, is defined as the use of game design elements in non-game contexts (22). Gamification of digital health and wellness technologies *via* gaming elements have been effectively used to optimize patient engagement, attention, motivation, and adherence to treatments and healthy lifestyle behaviors (22–24). For example, a clinically validated gamification health application *Rafi-Tone* was developed to promote effective inhaler techniques in asthmatic children, who may be at higher risk of getting sick from COVID-19, through a series of challenging games (25). Used by children and their parents, the application logs and monitors inhaler use and symptoms in an exciting way that increases patient adherence to treatment. Despite these successes, mixed results about the utility and long-term viability of gamified elements in digital health and wellness technologies has tempered enthusiasm for it. However, thoughtfully designed applications that build in habit forming goal-setting and self-monitoring to engender competence may empower users to seek desired behavior change outside of the reward system and thus increase the ecological validity of digital health solutions across different contexts and health conditions.

Digital health solutions must systematically incorporate behavioral health and learning theories and principles in their gamified mechanics and elements (e.g., challenges, chance rules, and tangible goals), components (discrete elements of a game), and features (rewards, user-facing components) to successfully increase engagement, uptake, long term use, and adherence to chronic disease management interventions. One successful use of gamification in chronic disease management is observed in the secondary prevention of diabetic complications through the optimization of glycemic control to lessen diabetic complications and improvement of patients' quality of life. This intervention used competence, a self-determination theory (SDT) concept associated with treatment adherence, as the primary behavioral and psychological driver in their diabetes digital health solution which aims to improve self-management behaviors through gamified and incremental goal setting. When patients' HbA1C targets are met they will feel they have mastered a task (26). Other digital health solutions have utilized gamified elements to increase social support and relatedness to improve chronic disease management (8, 27).

### Pantheoretical Approach: The Process of Contextualizing and Personalizing Digital Health Solutions and Gamified Elements

In addition to gamifying telemedicine and digital health solutions to increase uptake, long term use and adherence, we argue that contextualizing and personalizing these solutions are equally important. Contextualizing and personalizing chronic disease management experience provide a level of precision that can obviate barriers to healthcare and healthy lifestyle.

### Contextualizing Digital Health to Increase Adherence and Engagement

To increase uptake of telemedicine and digital health solutions requires contextualization (gamified programs developed across different settings and users) and personalization (*via* idiographic profiling), *via* the pantheoretical approach (21). Contextualizing telemedicine and digital health solutions through deep idiographic profiling (based on sociodemographic, clinical, and psychographic signatures of the individual) can optimize adherence and minimizes non-adherence to the desired health behavior. Developing an idiographic user phenotype/persona (e.g., cognitive and psychological factors) and incorporating contextual factors (e.g., race, economic status, neighborhood, geography, and social determinants of health) will increase the ecological utility, validity, and adaptability of digital health solutions across different settings and scenarios. A successful contextualization of a telemedicine and digital health solution can be seen in our digital social care navigator program which helps patients navigate contextual barriers to healthcare. Although this component is nascent, preliminary evidence from our work and others such as Aunt Bertha show promising results that social care navigation can improve engagement and adherence to digital health solutions and ultimately clinical outcomes, such as reduced re-admission rates by heart failure patients (26, 28).

### Personalizing Digital Health to Increase Adherence and Engagement

Personalizing the user's experience can also improve short- and long-term user engagement and adherence to telemedicine and digital health solutions. One effective strategy to implement and improve personalization is by determining the user's persona (based on social, medical, lifestyle, and personality domains) and understanding unique endogenous (e.g., motivation) and exogenous (e.g., poor access to care) barriers and facilitators to adherence. To ensure a deep understanding of users and how they will engage with a digital health solution, we must first develop an idiographic profile of users and then script their user journey based on their unique characteristics and needs. For example, below we describe how Livongo's AI-AI strategy effectively personalizes digital health solutions for diabetes and cardiovascular disease.

## Implementing a Digital Health Application for Chronic Disease Care Management Utilizing Pantheoretical Framework Strategies

**Figure 1:**
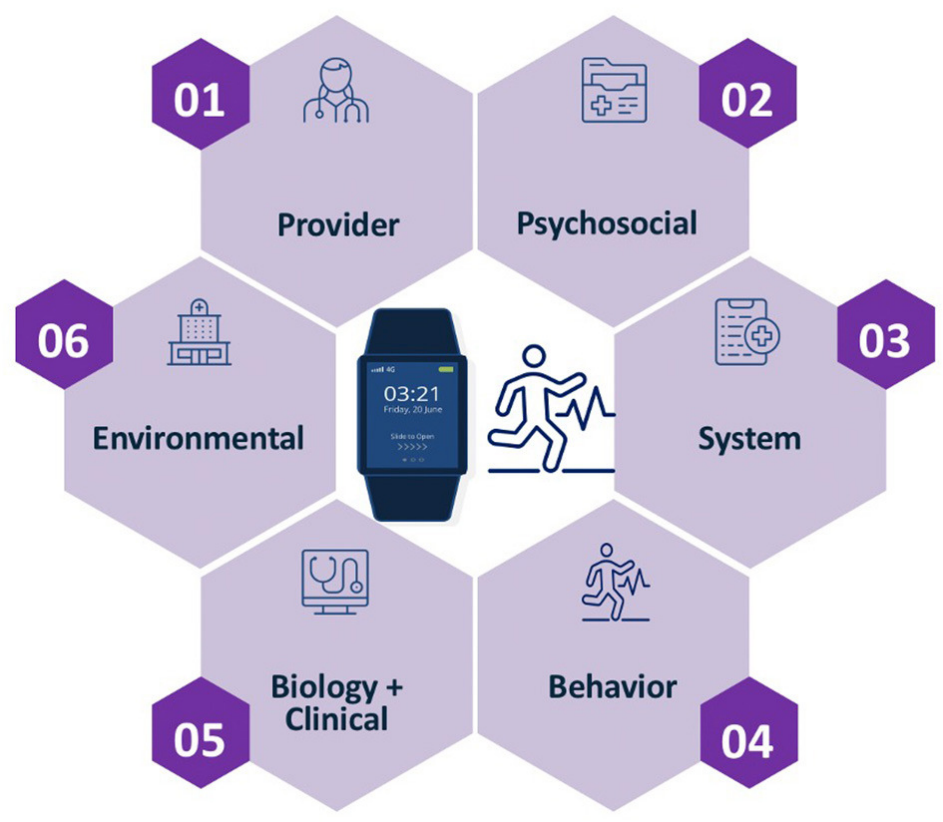
The use of a Pantheoretical framework to generate idiographic profiles of users to optimize digital health and wellness applications (21).

To implement digital health solutions utilizing the pantheoretical framework strategies, we suggest the following steps. First, assemble a multidisciplinary team that consists of providers, case managers, educational communications and technology/games for learning experts, behavioral scientists, data scientists, and health informaticians. Second, the multidisciplinary team must decide which digital health strategy and wellness solution appropriate through a mixed-method approach (using qualitative and quantitative data). Gamification of a digital health solution is not a one-size-fits-all approach and is one of many possible strategies to improve engagement and use of telemedicine and digital health solutions. If the patient is not an ideal candidate for telemedicine or digital health but whose medical care would be greatly improved with a digital solution, then the team can prepare and train the patient to accept and adhere to the digital solution, a training method described in a previous publication (21). Third, onboard patients with digital applications to familiarize them with the new application using simple and user-friendly experiential flows and interface elements to elicit baseline adherence to treatment and then ramped up for long term use. One strategy that has been successfully used to increase initial uptake of telemedicine and digital health solution is the use of onboarding quizzes and surveys. These tools can be used to develop idiographic and personalized phenotypes of users and tailored engagement strategies to optimize use. Fourth, monitor patient engagement behavior continuously through continuous data ingestion of patient's biological/clinical, behavioral, provider, system, psychosocial, and environmental characteristics (see Figure 1). Fifth, create personalized and precise algorithms (models of adherence) through the use of Artificial Intelligence and Machine Learning for each individual, generally executed through cloud computing to accommodate large streams of big data. Lastly, develop and continuously refine patients' personalized care support plan through mobile health solutions (nudges and education) over time.

## Practical Examples of Pantheoretical Approach

Gamifying, contextualizing, and personalizing digital health solutions to increase engagement and use has been successfully done in the management of several chronic diseases (29, 30). One of the most successful cases is Livongo's Diabetes and Cardiovascular disease package. The Livongo solution empowers people with diabetes or hypertension/pre-hypertension to monitor their blood pressure (BP) and glucose levels with cellular-connected BP monitor and glucometers, and to improve lifestyle habits with food and activity tracking. The Livongo package also includes real-time Health Nudges™ that deliver prompts when users are most receptive, human and digital coaching support, and medication optimization to increase adherence. Livongo captures and processes real-time data about barriers to self-management and through their algorithm provide customized educational materials and coaching tips to overcome these barriers. The process by which EMA and cardiovascular care adherence data will be ingested, analyzed, and visualized in real time to provide personalized curriculum and messages, based on participants' baseline and emerging data, highlights the depth of the pantheoretical approach. Personalization allows us to test what content to send (information), when to send it (time), how to send it (delivery), and to whom. This is known as Livongo's AI+AI (Augmented Intelligence and Artificial Intelligence) framework.

Livongo also uses machine learning to optimize adherence to self-management by building a dynamic and responsive system that can accurately anticipate and plan coaching resources to meet the participant's demands. Livongo uses historical participant data to train and refine a forecasting model that can accurately predict future demand. The Livongo AI-powered coaching system and the Applied Health Signals program are basically omniscient as they know a lot about the patient's needs and has detailed data on their medical progress, which boosts its ability to personalize care to patients. These features also considers all user interactions as opportunities to know them better and to more deeply personalize their medical journey. The algorithms and user journey are undergirded by well-established behavioral and social learning theories (31) and anchored by novel approaches such as the OBSSR-recommended mixed-method and SOBC approach (32), health literacy framework (14), patient-centered outcome research, and message tailoring through artificial intelligence. In sum, Livongo's gamified, contextualized, and personalized approaches, vis a vis the pantheoretcial approach, have been successfully used to improve glucose levels, weight loss, and reduce blood pressure (33–35).

The success of pantheoretical approaches and solutions like Livongo prompted several professional organizations like the American Heart Association (AHA) to adopt digital solutions in their larger strategic mission. The AHA incorporated digital health as a core interventional strategy to assess, diagnose, and improve several health outcomes, like blood pressure and atrial fibrillation (36–39). This roadmap has now expanded into other health conditions and programs, including heart failure, cardiac rehabilitation, stroke, diabetes, cholesterol, and mental well-being, through a network of technology solutions, including wearables, digital therapeutics, virtual and augmented reality designed to improve the digital health literacy, through knowledge, numeracy, navigation, communication, and decision making (40). This roadmap is ultimately the goal of improving healthy life expectancy in the US and around the world (41). The reality is that this goal cannot be accomplished without a robust digital health strategy.

## Limitations to Digital Chronic Disease Management Optimization

Despite the foregoing strengths of creating engaging digital health solutions that will be used long-term through gamification, contextualizing and personalizing strategies, a few methodological limitations must be considered before scaling. First, this approach requires deep and vast amounts of data and user experience engineering and cloud computing infrastructure to provide real-time analytics and engagement. Additionally, to fully optimize these approaches requires additional data and at this time, it is unclear how much data is actually needed to reach full optimization. Second, digital health solutions have inherent limitations such as: problems with access to devices and internet, low patient technology literacy and mastery of using digital tools, and lack of trust in the privacy and confidentiality of digital solutions. Regardless of these limitations, the enormous benefits of pantheoretical approaches to revolutionize digital health solutions and chronic disease management outweigh these limitations. To ensure telemedicine and digital health solutions actualize their potential and avoid the aforementioned limitations, developers must co-design and co-create digital health solutions with patients. Doing so will improve digital and technology health literacy, increase short- and long-term use of digital health solutions, and increase transparency of and trust in telemedicine and digital health solutions.

## Conclusion

The rapid pivot to digital tools has been an adjustment to patients and clinicians alike, serving as a lifeline to patients with chronic conditions. While digital solutions cannot completely replace all of the rich experience that comes with a face-to-face encounter, and fix healthcare inequities there are some advantages. Optimizing healthcare, especially for diverse and vulnerable patients with chronic diseases during this COVID era, will move healthcare and medicine to actualize its highest virtues. The unfortunate fragmentation and poor access to care replete in the current US healthcare system, amplified by COVID, can corrected through telemedicine and digital health solutions. This old system cannot operationally or economically meet the demands of the ballooning chronic disease burden in the US. Telemedicine and digital health solutions can transform an antiquated “one-size-fits-all” system in medicine and provide precise and personalized care to all. We argue that the fastest and most effective way of modifying this system and improving chronic disease patients adherence to care is to implement optimized digital health solutions. In order for telemedicine and digital health solutions to successfully increase use of, engagement with, motivation, and adherence to chronic disease self-management, telemedicine and digital health solutions must be personalized, contextualized, and rooted in behavioral science theories and gamification strategies through a pantheoretical approach.

## Data Availability Statement

The original contributions presented in the study are included in the article/supplementary material, further inquiries can be directed to the corresponding author/s.

## Author Contributions

AS: conceived and designed the manuscript. All authors met the ICMJE criteria for authorship, contributed equally in the subsequent preparation of the manuscript, wrote the initial draft, critically reviewed the manuscript, and reviewed and accepted the final version of the manuscript.

## Conflict of Interest

The authors declare that the research was conducted in the absence of any commercial or financial relationships that could be construed as a potential conflict of interest.
